# Electrodermal activity as an index of food neophobia outside the lab

**DOI:** 10.3389/fnrgo.2023.1297722

**Published:** 2024-01-03

**Authors:** Ivo V. Stuldreher, Erik Van der Burg, Sebastien Velut, Alexander Toet, Demi E. van Os, Haruka Hiraguchi, Maarten A. Hogervorst, Elizabeth H. Zandstra, Jan B. F. Van Erp, Anne-Marie Brouwer

**Affiliations:** ^1^Human Performance, Netherlands Organisation for Applied Scientific Research (TNO), Soesterberg, Netherlands; ^2^Human Media Interaction, University of Twente, Enschede, Netherlands; ^3^Brain and Cognition, University of Amsterdam, Amsterdam, Netherlands; ^4^Division of Human Nutrition and Health, Wageningen University & Research, Wageningen, Netherlands; ^5^Kikkoman Europe R&D Laboratory B.V., Wageningen, Netherlands; ^6^Consumer Science Insight, Unilever Foods Innovation Centre Wageningen, Wageningen, Netherlands; ^7^Human Machine Teaming, Netherlands Organisation for Applied Scientific Research (TNO), Soesterberg, Netherlands; ^8^Donders Institute for Brain, Cognition and Behaviour, Radboud University, Nijmegen, Netherlands

**Keywords:** electrodermal activity, food neophobia, food, arousal, anticipatory response, music festival

## Abstract

**Introduction:**

Understanding how food neophobia affects food experience may help to shift toward sustainable diets. Previous research suggests that individuals with higher food neophobia are more aroused and attentive when observing food-related stimuli. The present study examined whether electrodermal activity (EDA), as index of arousal, relates to food neophobia outside the lab when exposed to a single piece of food.

**Methods:**

The EDA of 153 participants was analyzed as part of a larger experiment conducted at a festival. Participants completed the 10-item Food Neophobia Scale. Subsequently, they saw three lids covering three foods: a hotdog labeled as “meat”, a hotdog labeled as “100% plant-based”, and tofu labeled as “100% plant-based”. Participants lifted the lids consecutively and the area-under-the-curve (AUC) of the skin conductance response (SCR) was captured between 20 s before and 20 s after each food reveal.

**Results:**

We found a significant positive correlation between food neophobia and AUC of SCR during presentation of the first and second hotdog and a trend for tofu. These correlations remained significant even when only including the SCR data prior to the food reveal (i.e., an anticipatory response).

**Discussion:**

The association between food neophobia and EDA indicates that food neophobic individuals are more aroused upon the presentation of food. We show for the first time that the anticipation of being presented with food already increased arousal for food neophobic individuals. These findings also indicate that EDA can be meaningfully determined using wearables outside the lab, in a relatively uncontrolled setting for single-trial analysis.

## Introduction

The willingness to explore novel and unfamiliar foods can be captured through the 10-item food neophobia scale (Pliner and Hobden, 1992). According to this scale, individuals with high scores are generally more hesitant to try or buy new foods compared to individuals with low scores (Arvola et al., 1999). Food neophobia has been linked to reduced dietary variety and quality, and it can be a barrier to shift toward novel or unfamiliar healthier and more sustainable food products (Jaeger et al., 2017). Therefore, it is important to understand how food neophobia affects food choice and food experience.

One approach is to simply ask participants how they experience a certain food stimulus. Studies using such explicit ratings have repeatedly shown a negative association between food neophobia and hedonic liking of novel food stimuli (for a systematic review, see: Rabadán and Bernabéu, 2021). The rejection of and decreased hedonic liking of food through food neophobia may be caused by increased arousal (Jaeger et al., 2021; Costa et al., 2023). Foods with high flavor intensity, from other cultures, perceived as dangerous or that are novel—thus with high arousal characteristics—have the strongest negative relationship between food neophobia and hedonic liking (Jaeger et al., 2021).

Explicit measures of food experience may be augmented by physiological measurements, such as electrodermal activity (EDA). EDA reflects the changes in electric properties of the skin and is a direct marker of activation of the sympathetic branch of the autonomic nervous system (Boucsein, 2012), and due to its unique innervation, a sensitive marker of arousal.

A small number of studies using physiological measures have shown that food neophobic individuals are typically more aroused and more attentive when observing food images. For instance, Raudenbush and Capiola (2012) reported that heart rate, EDA and respiration during the presentation of a variety of food images were significantly increased in food neophobic individuals compared to controls, indicating higher arousal in the former group than the latter. Using electroencephalogram (EEG), Stuldreher et al. (2023) found evidence that attention to food pictures increases with food neophobia. More specifically, they reported a positive correlation between the food neophobia score and the late positive potential (LPP) amplitude when the participants observed food images. The LPP is an event-related potential component over the parietal cortex that is a marker of attentional resource allocation, which is for instance also larger when viewing affective compared to neutral images (Schupp et al., 2000). Interestingly, the association between the LPP amplitude and food neophobia was not only present when the participants observed novel food images, but also when they observed familiar food images, indicating that food neophobia affects attention more broadly than only toward novel food. In the same paper, Stuldreher et al. (2023) also showed a positive correlation between food neophobia and another measure of attention, interpersonal physiological synchrony in EEG, when watching a movie on novel food. More evidence for a generally more aroused state of food neophobic individuals during food experience comes from a recent online study by Jaeger et al. (2023). In their study, over 7,000 individuals from four different countries rated the arousal of a series of food names (such as “vegetable stir-fry”, “Moroccan carrot salad”, “Pizza with tomato & cheese” or “Ox tongue noodle soup”). Consistent with the Schupp et al. study, the mean arousal rating increased with the food neophobia of participants (Jaeger et al., 2023).

Physiological responses, particularly electrodermal responses, have been studied using visual and auditory stimuli, while its use in response to chemosensory stimuli—or real foods—is limited. This is noteworthy because initial findings suggest that electrodermal responses to taste and smell are significantly more pronounced compared to responses triggered by other sensory stimuli (Glass et al., 2014). As for food related pictures, EDA and heart rate responses are more pronounced for unliked compared to liked stimuli (Kaneko et al., 2019; Lagast et al., 2020) and response patterns also differ between novel and familiar foods (Brouwer et al., 2017). Taken together, there is ample evidence using several methodologies that arousal and attention increase with food neophobia when *confronted* with (familiar or unfamiliar) food related stimuli (like images, and even names).

To date, it remains unclear whether attention and arousal increase with food neophobia using real food samples. Moreover, the vast majority of studies investigating the relation between arousal or attention and food neophobia have been conducted in controlled laboratory environments using high-end equipment. In these studies, the dependent variable was typically derived after averaging the responses over multiple trials of stimulus presentation. The generalizability of these findings to real-world scenarios where people make food choices and consume food is an important, yet underexplored question. Given the natural context, including body movements and higher chances of electrode motion artifacts, it is important to carefully preprocess the possibly noisy EDA signals. In the present study, we examined whether it is feasible to measure arousal using EDA in an out-of-lab context where participants observe a small number of real food items, analysis is done on each single item as opposed to repeated exposures to food images, and recordings are performed under relatively noisy, varying conditions. The corresponding research questions is: how can arousal in relation to food neophobia be reliably measured in an out-of-lab setting? If we are able to measure arousal in a reliable manner, then we expect to observe a positive correlation between arousal and food neophobia.

If an increased arousal is indeed inherently associated to the rejection of food due to food neophobia, as stated in the arousal hypothesis by Jaeger et al. (2021), increased arousal as indexed by an increased EDA might precede the actual presentation and thus perception of foods. This would also be in line with the increased attention of food neophobic individuals upon the presentation of food stimuli that we found previously (Stuldreher et al., 2023), as arousal and attention are closely coupled (Critchley, 2002). To the best of our knowledge, it has not been confirmed whether the level of arousal relates to the level of food neophobia in anticipation of the perception of food stimuli. Our second research question therefore is: how is the anticipatory response in EDA related to food neophobia?

## Methods

### Participants

Two hundred and forty participants (138 females) with a mean age of 31.8 ± 10.8 years, were recruited at Lowlands (https://lowlands.nl/), a three-day music festival in the Netherlands (August 19–21, 2022). All participants were naïve as to the purpose of the experiment and signed an informed consent before partaking. The experiment was approved by the TNO institutional review board (reference number: 2021-071).

### Materials

EDA was recorded at 32 Hz using EdaMove 4 wearables (Movisens GmbH, Karlsruhe, Germany). The data were recorded from the palmar surface of the non-dominant hand using two solid gelled Ag/AgCl electrodes (MTG IMIELLA electrode, MTG Medizintechnik, Lugau, Germany, W55 SG, textured fleece electrodes, 55 mm diameter). In a previous study we have found good correspondence between these sensors an high-end laboratory equipment (Borovac et al., 2020).

### Stimuli

**Figure 1:**
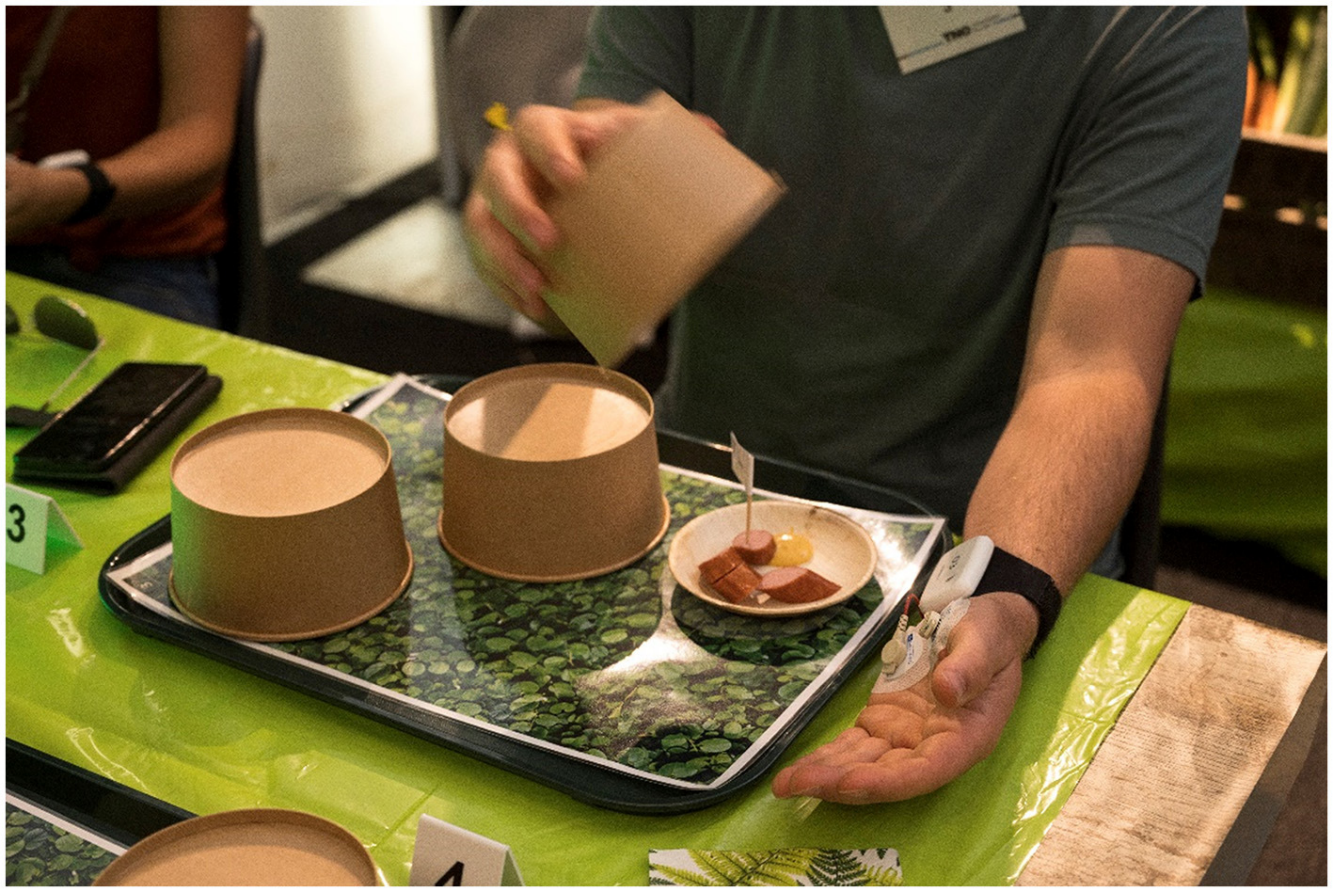
The three foods were presented on separate plates, each covered by a lid on a single tray placed in front of the participants. Upon instruction the participants lifted the lid of the plates one by one.

Participants were presented with three different foods, being a plant-based hotdog (brand: The Vegetarian Butcher), another plant-based hotdog (brand: the Vegetarian Butcher) *or* a meat hotdog (brand: Unox), and tofu (brand: Albert Heijn). The hotdogs were served warm and combined with a hotdog sauce (brand: Calvé). The tofu was served cold and combined with soy sauce (brand: Kikkoman). As is shown in Figure 1, the three bites were presented on separate plates, each covered by a lid on a single tray that was placed in front of the participant.

Each snack was presented with a white miniflag with the text “Meat” or “100% plant-based”. The plates with hotdogs were placed in the bottom-left and bottom-middle of the tray, the plate with tofu was placed in the top-right of the tray. The foods were to be revealed from left to right. The order of the hotdogs labeled as “Meat” and “100% plant-based” were counterbalanced across participants.

### Procedure

The experiment took place on three consecutive days between 12:00 and 20:00 h at the Lowlands music festival. The intervention we describe here was part of a larger experiment on the influence of information and context on people's food choice and tasting experience regarding plant-based meat vs. animal meat. About every hour, a group of eight volunteers participated in the experiment. The EdaMove 4 sensors were attached to the non-dominant hand, the participants were given a smartphone and they were then seated in the “pitch-tent”. Here, participants completed a questionnaire regarding their attitudes and behavior toward food and completed the food neophobia questionnaire using the smartphone (Pliner and Hobden, 1992). This questionnaire consists of ten statements, for which a rating on a 7-point Likert scale, ranging from “strongly disagree” to “strongly agree”, can be given. The outcome—a score from 10 to 70—indicates the willingness to try novel foods. High scores indicate high food neophobia, meaning unwillingness to try new foods, while low scores indicate enthusiasm to try novel food. Participants wore a headphone and were presented with a five-minute video pitch on a large TV screen. The pitch was given by Prof. Erik Scherder, a Dutch neuroscientist known from the Dutch television. The pitch was on either the consumption of sustainable food and its effect on the environment and health, or on the effects of movement on brain health. After the pitch, participants answered questions concerning the pitch using the smartphone. Data obtained during and immediately after the pitch are reported elsewhere. Participants were then guided to the second tent, which was either setup in a sustainable, meat or neutral context. Data exploration and statistical tests indicated that these different conditions did not significantly impact EDA responses as described here. An experiment leader instructed participants that they were to be presented with two foods consecutively. The experiment leader instructed participants that they could lift the lid of the leftmost plate on their tray after a five second countdown, and observe the contents of the plate for 20 s. After this time and another five second countdown participants could lift the lid of the second, middle plate on their tray and observe the contents for 20 s. After each countdown, the experiment leaders tapped the EdaMove 4 device around their wrist to send a trigger that could be used to link participants' electrodermal response to the food reveal. After presentation of both hotdogs, participants were asked which of the two hotdogs they would like to taste (the “Meat” or “100% plant-based”), how hungry they were, how much they were looking forward to eating the hotdog and how tasty they thought that the hotdog would be. Participants could then taste as many pieces of their chosen hotdog as they wanted, up to a maximum of four pieces. Directly after consumption, participants answered several questions on liking and taste perception of the chosen hotdog. Then, the experiment continued with the presentation of the tofu served with soy sauce. As with the hotdogs, after a five-second countdown the experiment leader tapped their EdaMove 4, and participants could lift the lid of their plate. After roughly 20 s, participants were instructed to first smell the soy sauce, taste and rate the tofu in combination with the soy sauce following questions on the smartphone. Participants could then remove their EdaMove 4 device and finished the experiment. In total, one run of the experiment took 20 min.

### Analysis

The fully anonymous data and scripts used for analysis are available online at https://osf.io/j9ukc/. Data were analyzed using MATLAB R2021a (Mathworks, Natick, MA, USA). EDA recordings were first processed to extract the EDA of individual participants. EDA was epoched to the onset of the three food reveals using the triggers sent by the experiment leader. Artifacts in EDA were removed following (Thammasan et al., 2020). First, parts of the data where the signal was below 1 μS were marked as artifactual, as this indicates a disconnected electrode. Second, parts of the data surrounding data marked as artifactual or in between two segments of data marked as artifactual were also marked as artifactual. The marked data were replaced by NaN values. If more than one-third of the EDA of a participant recorded during the 20 s prior to the food reveal until 20 s after the food reveal were marked as artifactual, this participant was removed from further analyses.

**Figure 2:**
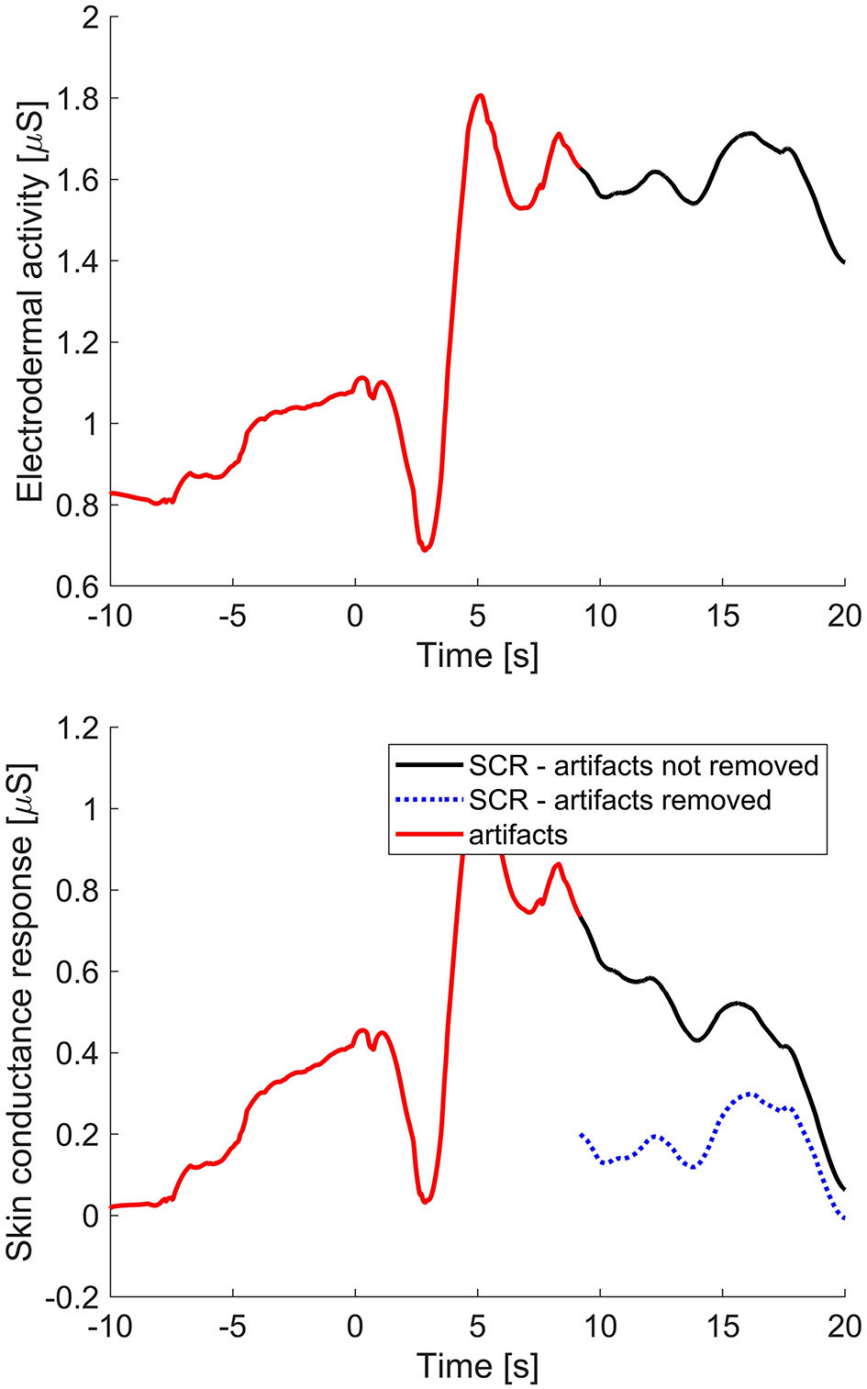
Effect of removal of artifacts in the electrodermal activity (EDA) on the skin conductance response (SCR). The **top** window shows the EDA of a participant (black line) in which artifactual samples have been detected (red line). The **bottom** window shows the effect of two ways of dealing with the artifact on the resulting SCR. If the artifact is left in EDA data that is decomposed to obtain the SCR we obtain the black line, after which the artifacts (in red) can be removed, but the signal is still corrupted (i.e. higher than it should be). If we decompose the signal parts in between artifactual segments separately, thereby not including artifactual periods in the decomposition, we obtain the blue dotted line, which represents a cleaner signal.

The EDA of the remaining participants were then smoothed using a third order Savitzky-Golay filter to remove quantization noise (Thammasan et al., 2020). EDA was then further processed to obtain the phasic component, also known as the skin conductance response (SCR). To do so, we used continuous decomposition analysis as implemented in the Ledalab toolbox for MATLAB (Benedek and Kaernbach, 2010). Note that performing continuous decomposition analysis on the EDA signal without removing artifacts first strongly impacts the result of the analysis of the parts surrounding artifactual data (see Figure 2). We therefore performed the continuous decomposition analysis separately on all segments in between parts of the data marked as NaN.

**Figure 3:**
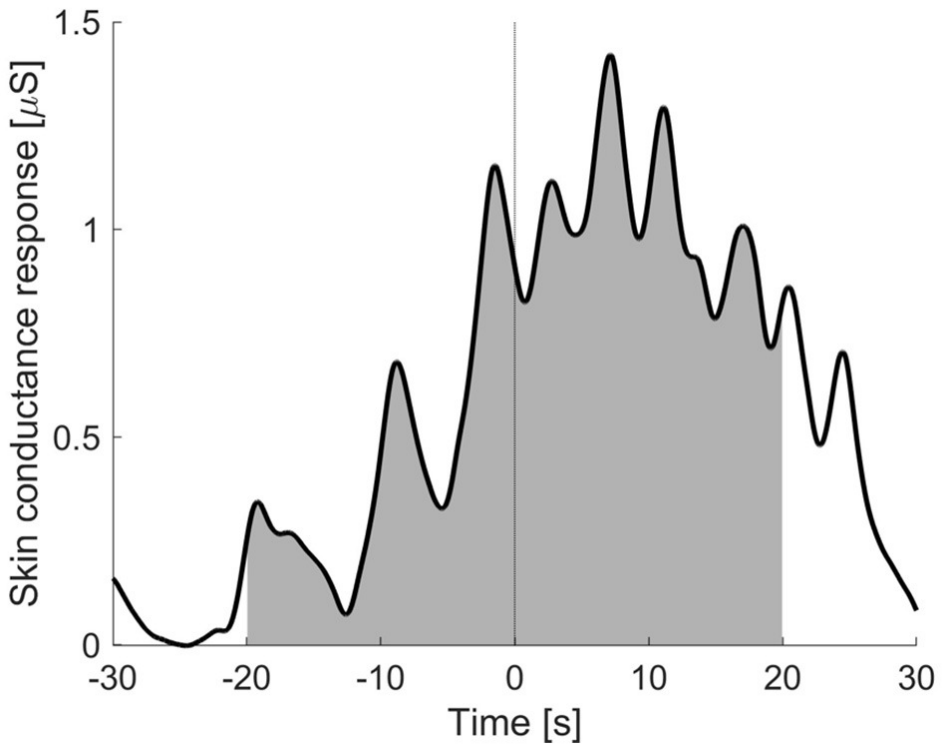
Example of the area under the curve (AUC) of the skin conductance response in the period ranging from 20 seconds prior to 20 s after the food reveal.

We then extracted the area under the curve (AUC) of the skin conductance response from 20 s prior to each food reveal up to 20 s after each food reveal, as depicted in Figure 3. The AUC was corrected for missing data by dividing all AUC values by the number of valid samples. We chose this method in contrast to other metrics such as response amplitude as through this method we can capture the arousal during the entire period around the food reveal instead of only capturing the arousal at one time point. We tested for an association of the AUC of the SCR during the three food reveals (hotdog one, hotdog two and tofu) with food neophobia using Spearman correlation analysis. The Spearman correlation was selected instead of Pearson as due to the positive skew in SCR variables we did not expect a linear relationship between AUC of SCR and food neophobia (Boucsein, 2012). We repeated these analyses using only the AUC of SCR prior to the food reveal (i.e., *t* < 0) and using only the AUC of SCR after the food reveal (i.e., *t* > 0). This analysis enabled us to understand whether any potential correlations are mainly driven by the anticipation of being presented with food, or the response of seeing this food.

For visualization purposes, we split the participant group in food neophobic and food neophiliac subgroups using a median split on the food neophobia scores.

## Results

### Data loss

EDA recordings of 10 participants were lost as data of one sensor were unintentionally deleted. Data of 68 additional participants were lost due to missing food reveal markers, such that EDA responses could not be linked to any food reveal. Data of four additional participants were lost due to sensor failure. Data of nine additional participants were removed based on our artifact removal criteria. In total, data of 153 participants were used for further analyses. From three of these participants, 9, 12, and 23 percent of data, respectively, were removed based on our artifact removal criteria.

### SCR and food neophobia

**Figure 4:**
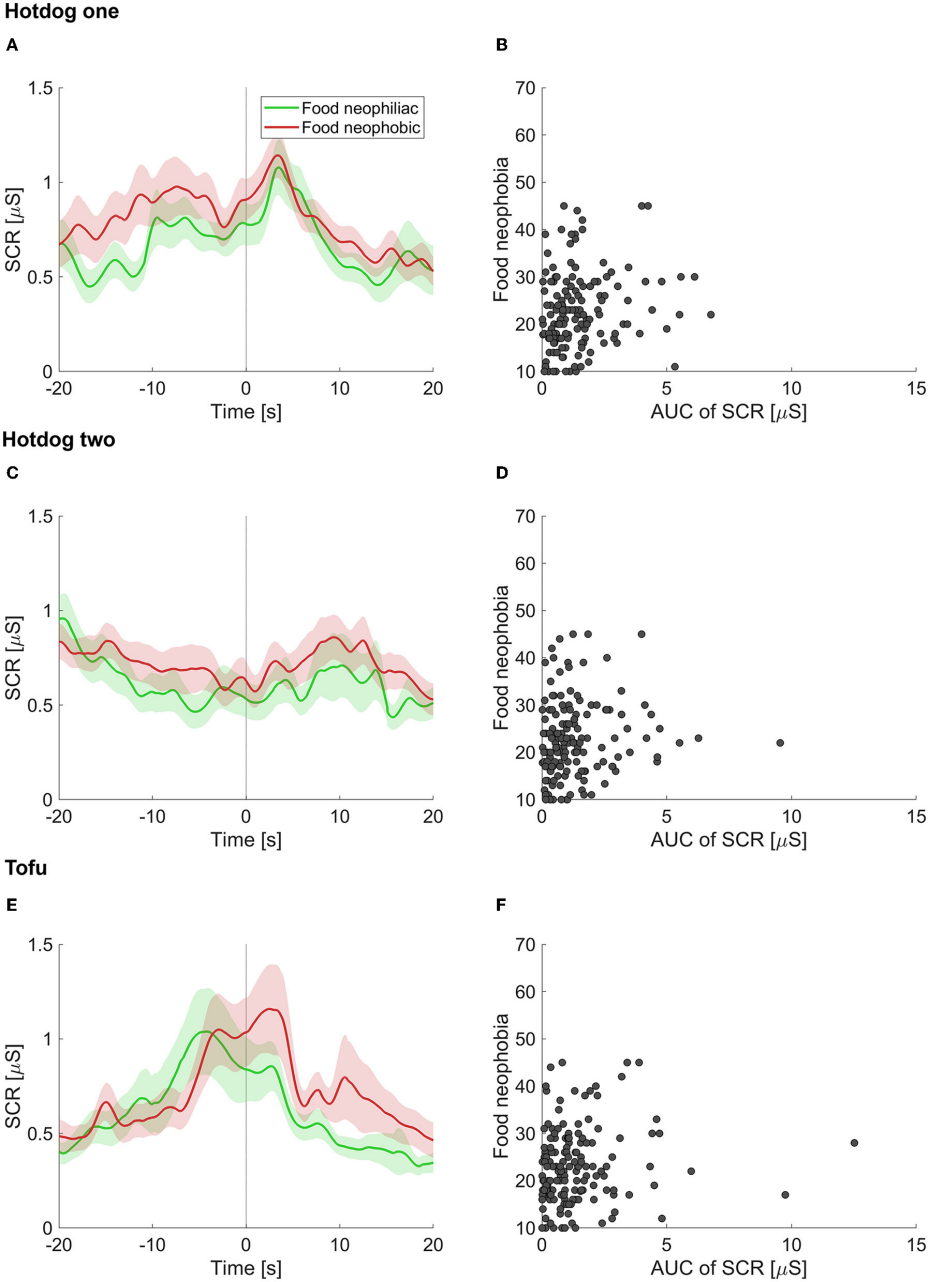
Results of the experiment. **(A, C, E)** Skin conductance response (SCR) over time for food neophiliac (green) and food neophobic (red) participants. The time is relative to the food reveal (0 ms). The shaded area corresponds to the standard error of the mean across participants. **(B, D, F)** Food neophobia score as a function of the area under the curve (AUC) of SCR for each individual participant. Upper, middle and lower panels correspond to hotdog one, hotdog two, and to tofu, respectively.

The mean food neophobia score of our sample of participants was 22.8 (SD: 8.0). Figures 4A, C, E, depicts the mean SCR over time for food neophiliac and food neophobic participants upon presentation of hotdog one, hotdog two and the tofu, respectively. The SCR increases before the food reveal, which is especially clear for food neophiliac participants upon the presentation of hotdog one. The figures further indicate that for hotdog one and hotdog two the SCR is higher for relatively food neophobic than food neophiliac participants around the time of the food reveal. Figures 4B, D, F illustrates the food neophobia score as a function of the AUC for SCR for each participant. Positive, significant Spearman correlations were found between the food neophobia score and the AUC for SCR corresponding to the presentation of the first hotdog (*r* = 0.19, *p* = 0.018) and second hotdog (*r* = 0.16, *p* = 0.047); only a trend was found around the presentation of the tofu (*r* = 0.14, *p* = 0.090).

For the presentation of the first hotdog, the correlations were also significant when using only the SCR data prior to the food reveal (*r* = 0.18, *p* = 0.025) and SCR data after the food reveal (*r* = 0.17, *p* = 0.037). For the presentation of the second hotdog, the correlation was only significant when using the SCR data prior to the food reveal (*r* = 0.18, *p* = 0.031), but not when using the SCR after the food reveal (*r* = 0.14, *p* = 0.075). For the presentation of the tofu, the correlations were both not significant (prior: *r* = 0.10, *p* = 0.195, post: *r* = 0.14, *p* = 0.089).

## Discussion

In the current study we investigated whether we could capture arousal using wearable EDA sensors after one exposure upon the presentation of small pieces of foods (two hotdogs followed by tofu) outside the lab. We found a significant positive correlation between the SCR and food neophobia for both hotdogs, and a similar trend for tofu, indicating that the SCR response increased with increasing food neophobia scores. The significant correlations were also observed when focusing on the EDA response prior to food reveal. This indicates that the anticipation of being presented with food is enough to increase arousal for food neophobic individuals, and that observing the food item is not a prerequisite for observing an increased arousal. This is in line with the arousal hypothesis, which states that the increased arousal is inherently associated with the relation between food neophobia and food rejection (Jaeger et al., 2021). It is also in line with our previous study (Stuldreher et al., 2023), where we showed that food neophobic individuals were more attentive to food images in general (i.e., regardless the familiarity of the food item). As it is food in general that strongly draws attention in food neophobics, and as attention and arousal are related (Critchley, 2002), it is not surprising that we find that food neophobic individuals are already more aroused than food neophiliac individuals prior to the actual food is revealed (i.e., an anticipatory response).

One could argue that our findings are driven by general anxiety. Anxiety is said to play a role in food neophobia, with numerous studies showing a significant relation between measures of food neophobia and anxiety (Pliner and Hobden, 1992; Galloway et al., 2003; Agovi et al., 2022). Food neophobia can cause attentional biases toward pictures of food (Maratos and Staples, 2015), though this effect is not always found (Agovi et al., 2022). Similarly, anxiety traits can cause attentional biases and increased EDA toward novel stimuli in general (Rabavilas, 1987). Though we did not explicitly test it in the current study, we believe that our results are specific to food neophobia and not driven by the relation between general anxiety and novelty alone. Food neophobic individuals were shown to have an attentional bias and increased EDA in response to food-related stimuli (Raudenbush and Capiola, 2012; Stuldreher et al., 2023), while such effects have not been found in response to non-food stimuli (Raudenbush and Capiola, 2012). This suggests that it is not the association between anxiety and increased EDA toward novel stimuli that drives increased EDA in our study.

Whereas we found a significant correlation between the AUC for SCR and food neophobia for the first two hotdogs, no such correlation was observed for the third food item (i.e., the tofu). We previously mentioned that food neophobic individuals also show increased arousal and attention to familiar foods, such that increased arousal is not dependent on food familiarity. This however only holds when the to be presented stimulus is unknown. If a familiar food is expected, the anticipatory response may be reduced as there is less expectation of an unfamiliar food. A possible explanation for the absence of a significant effect for tofu therefore is that food neophobic individuals were reassured by the relatively familiar hotdogs by the time that the third food was going to be revealed, such that their response did not significantly exceed that of food neophiliac individuals. Such habituation effects have previously been reported for EDA (Verastegui-Tena et al., 2018). Also relevant in this respect is that none of the participants in our sample scored high on the food neophobia scale (all scores 45 or lower). The age of our participants coincides with the adult age group that generally shows the lowest food neophobia (as recorded in Ireland; Hazley et al., 2022). The population that is attracted to festivals such as where we conducted the experiment can also be expected to be open-minded toward exploring new food. It is therefore not surprising that our population showed lower food neophobia scores compared to other studies with similar age characteristics (Predieri et al., 2020; Hazley et al., 2022). In addition, as it was clear to potential participants that our experiment was about food there may have been self-selection bias, where individuals with higher levels of food neophobia did not choose to participate. The fact that even with limited variation in food neophobia scores of participants we found this relation vouches for the robustness of the association. Still, our findings should be confirmed in populations with more varying levels of food neophobia.

From a methodological perspective it is worth to note that without removing artifactual data parts the correlation between EDA and food neophobia was not reliable, indicating that it is important to remove artifacts when measuring EDA outside to lab for use in single-trial analysis. Note that in general the proportion of variance in food neophobia that is explained by the AUC of SCR is expected to be modest. There are many factors that affect the magnitude of the EDA response, such as temperature, physical activity, electrode movement, electrode placement besides the already complex relationship between emotional arousal and EDA (Kapp et al., 2014). Similarly, food neophobia scores may be correlated with arousal when presented with food-related items, that could be captured by EDA, but is also affected by many other factors, such as individuals' traits and states, social influence, information on the types of food, and many more (Pliner and Salvy, 2006).

In conclusion, we here found that food neophobia is positively associated with arousal upon a single presentation of small pieces of real food as captured using wearable EDA sensors outside the lab. Interestingly, the anticipation of being presented with food is enough to increase arousal in food neophobic individuals, and that physically observing food is not a prerequisite. Our findings also indicate that EDA can be reliably and meaningfully determined using wearable sensors in a relatively uncontrolled environment outside the lab for single-trial analysis.

## Data availability statement

The datasets presented in this study can be found in online repositories. The names of the repository/repositories and accession number(s) can be found below: https://osf.io/j9ukc/.

## Ethics statement

The studies involving humans were approved by TNO Institutional Review Board. The studies were conducted in accordance with the local legislation and institutional requirements. The participants provided their written informed consent to participate in this study.

## Author contributions

IS: Data curation, Formal analysis, Methodology, Software, Visualization, Writing—original draft. EV: Conceptualization, Formal analysis, Investigation, Methodology, Software, Writing—review & editing. SV: Investigation, Software, Writing—review & editing. AT: Conceptualization, Investigation, Methodology, Writing—review & editing. DO: Writing—review & editing. HH: Investigation, Writing—review & editing. MH: Investigation, Writing—review & editing. EZ: Conceptualization, Funding acquisition, Investigation, Methodology, Resources, Writing—review & editing. JV: Conceptualization, Investigation, Methodology, Resources, Supervision, Writing—review & editing. A-MB: Conceptualization, Funding acquisition, Investigation, Methodology, Project administration, Resources, Supervision, Writing—review & editing.
